# SARS-CoV-2 Infection-and mRNA Vaccine-induced Humoral Immunity among Schoolchildren in Hawassa, Ethiopia

**DOI:** 10.3389/fimmu.2023.1163688

**Published:** 2023-06-15

**Authors:** Yared Merid, Wondwosen Tekleselasie, Emnet Tesfaye, Anteneh Gadisa, Dessalegn Fentahun, Alegntaw Abate, Aynalem Alemu, Adane Mihret, Andargachew Mulu, Tesfaye Gelanew

**Affiliations:** ^1^ College of Medicine and Health Sciences, Hawassa University, Hawassa, Ethiopia; ^2^ Armauer Hansen Research Institute, Addis Ababa, Ethiopia; ^3^ Hawassa College of Health Sciences, Hawassa, Ethiopia

**Keywords:** SARS-CoV-2, RBD, antibody, COVID-19, BNT162b2 vaccine, dose, schoolchildren, variant

## Abstract

**Background:**

With the persisting low vaccination intake, particularly in children of low-and middle-income countries (LMICs), seroepidemiological studies are urgently needed to guide and tailor COVID-19 pandemic response efforts in schools and to put mitigation strategies in place for a future post-pandemic resurgence. However, there is limited data on SARS-CoV-2 infection-induced and vaccine-induced humoral immunity in schoolchildren in LMICs, including Ethiopia.

**Methods:**

As the spike receptor binding domain (RBD) is the major target for neutralization antibodies and useful to predict the correlates of protection, we used an in-house anti-RBD IgG ELISA to assess and compare infection-induced antibody response at two-time points and BNT162b2 (BNT) vaccine-induced antibody response at a one-time point in schoolchildren in Hawassa, Ethiopia. In addition, we measured and compared the levels of binding IgA antibodies to spike RBD of SARS-CoV-2 Wild type, Delta, and Omicron variants in a small subset of unvaccinated and BNT-vaccinated schoolchildren.

**Results:**

When we compare SARS-CoV-2 infection-induced seroprevalences among unvaccinated school children (7-19 years) at the two blood sampling points with a 5-month interval, we observed an over 10% increase, from 51.8% (219/419) in the first week of December 2021 (post-Delta wave) to 67.4% (60/89) by the end of May 2022 (post-Omicron wave). Additionally, we found a significant correlation (*p* = 0.001) between anti-RBD IgG seropositivity and a history of having COVID-19-like symptoms. Compared to the levels of SARS-CoV-2 infection-induced anti-RBD IgG antibodies before vaccination, higher levels of BNT vaccine-induced anti-RBD IgG antibodies were observed even in SARS-CoV-2 infection-naïve schoolchildren of all age groups (*p* = 0.0001). Importantly, one dose of the BNT vaccine was shown to be adequate to elicit a strong antibody response in schoolchildren with pre-existing anti-RBD IgG antibodies comparable to that of SARS-CoV-2 infection-naive schoolchildren receiving two doses of BNT vaccine, suggesting a single dose administration of the BNT vaccine could be considered for schoolchildren who had prior SARS-CoV-2 infection when a shortage of vaccine supply is a limiting factor to administer two doses irrespective of their serostatus. Despite the small sample size of study participants, the BNT vaccine is shown to be immunogenic and safe for schoolchildren. Irrespective of schoolchildren’s vaccination status, we observed a similar pattern of significantly higher levels of IgA antibodies to Delta-RBD than to Omicron-RBD (*p* < 0.001) in a randomly selected subset of schoolchildren, yet comparable to Wuhan-RBD, suggesting these schoolchildren were more likely to have had SARS-CoV-2 infection with Delta variant. Additionally, we noted a broader IgA antibody reactivity to SARS-CoV-2 variants in vaccinated schoolchildren with prior SARS-CoV-2 infection, supporting the superiority of hybrid immunity.

**Conclusion:**

Our serological data indicate a significant increase in SARS-CoV-2 seroprevalence in children at a post-Omicron five-month follow-up compared to a post-Delta enrolment. Despite the small sample size of study participants, the BNT vaccine is shown to be immunogenic and safe for schoolchildren. Hybrid immunity would likely provide a broader humoral immunity against Wuhan strain, Delta, and Omicron variants than natural infection or vaccination alone does. However, future longitudinal cohort studies in SARS-CoV-2-naïve and COVID-19-recovered schoolchildren receiving the BNT vaccine are needed for a better understanding of the kinetics, breadth, and durability of BNT vaccine-induced multivariant-cross reactive immunity.

## 1 Introduction

Children are usually more susceptible to viral infection than adults, however, the incidence of SARS-CoV-2 infection and fatalities in children had been reported to be very low (1, 2). Recently, however, after the emergence of the immune-evasive SARS-CoV-2 Omicron variant and subvariants, the number of infections and hospitalizations has increased in children of age 0–19 years (3, 4). Very recently, a meta-analysis of global seroprevalence studies indicates increasing seropositivity and symptomatic cases in children in the post-Omicron era (5), warranting the need to consider increasing testing and vaccination. With the persisting low vaccination intake, particularly in children of low-and middle-income countries (LMICs), seroepidemiological studies are urgently needed to guide and tailor COVID-19 pandemic response efforts in schools and to put mitigation strategies in place for future post-pandemic resurgences. However, there is limited data on the prevalence of post-SARS-CoV-2 infection antibodies in schoolchildren from resource-limited settings, including Ethiopia.

Although vaccination against SARS-CoV-2 infection is proven to be safe and effective for adults in the pre-Omicron era at least for six months (6), children remain the largest unvaccinated group worldwide. This pre-Omicron COVID-19 vaccine disparity situation between adults and children is due to the lower risk of COVID-19 disease in children (7), concerns about vaccine safety, and parental hesitancy (8). After the emergence of the Omicron variant and subvariants, there has been an increasing number of children requiring critical care in both developed and developing countries (4). Importantly, there was a growing concern that the reopening of schools would accelerate SARS-CoV-2 school transmission (9) and the emergence of new variants among children in the future in the absence of child COVID vaccination (10). These factors led to the implementation of vaccination for children globally as of late August 2021 (10). Immunogenicity and safety studies from developed countries have proven the safety and effectiveness of mRNA-based COVID-19 vaccines, including the Pfizer-BioNTech BNT162b2 vaccine (hereafter designated as BNT) against different variants, including the Omicron variant in children (11–13).

Although COVID-19 vaccines have become more available to Africans, including children through the African Vaccine Acquisition Task Team of the African Union and the World Health Organization (WHO)-led COVAX consortium (14), there is limited or no local evidence regarding vaccine-induced immune response among pediatric populations in Africa, particularly in Ethiopia. The generation of virus-specific antibodies which neutralize or block infectivity is the most consistent correlate of protective immunity for multiple infections and vaccines (6, 15, 16). Given the role of SARS-CoV-2 receptor binding domain (RBD) in SARS-CoV-2 entry to host cells via angiotensin-converting enzyme 2 (ACE2) and anti-RBD antibodies’ role in blocking virus binding to host receptors, measuring the level of anti-RBD IgG antibodies is considered as a proxy marker for virus neutralization, and indirect indication for the effectiveness of a vaccine against SARS-CoV-2 (17, 18). In this study, we sought to assess and compare SARS-CoV-2 infection-induced and BNT vaccine-induced humoral immunity in the longitudinal cohort of schoolchildren in Hawassa and its surrounding area using in-house anti-SARS-CoV-2 RBD ELISA.

Additionally, considering our first round (baseline) and the second round (5-month follow-up) blood sample collections were succeeded by the third and fourth Ethiopian pandemic waves due to the Delta and Omicron variants, respectively (19), we quantified and compared the levels of binding IgA antibodies to Delta-RBD and Omicron-RBD in comparison to Wuhan-RBD in a subset of randomly selected serum panels that had detectable anti-RBD IgG antibodies.

## 2 Materials and methods

### 2.1 Study area

**Figure 1:**
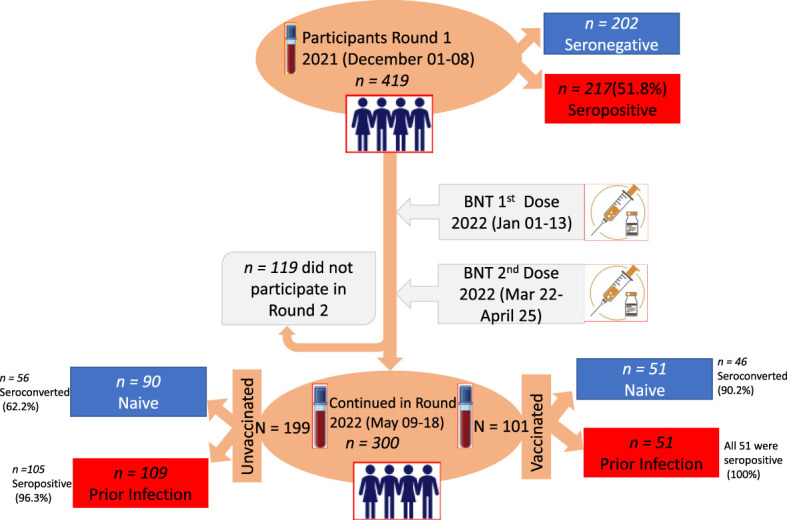
Study design, time points for the administration of first dose (1d) and second dose (2d) vaccination, and saliva and blood samples collected with the corresponding number (n) of participants. The two time points at a 5-month interval selected for serum and saliva sample collection are indicated on the vertical time axis with blood collection tubes.

A two-time-point correlational study (**Figure 1**) was carried out in six schools (**Table 1**) with both school types (elementary and secondary schools) in Hawassa City and Tula Town. Hawassa City is located 273 km away from the capital city of Ethiopia, Addis Ababa, whereas Tula Town is located 10 km away from Hawassa City. Except for Tula, all the remaining five study-participating schools are in Hawassa City, an urban setting. The participating schools were selected purposefully considering the geographic location in the city and the number of schoolchildren enrolled in the schools, while the participating schoolchildren were randomly selected from each school using a proportionate sampling method (**Table 1**).

**Table 1:** Enrolment seroprevalence of anti-SARS-CoV-2 antibodies and RT-PCR positivity rates in six schools in Hawassa, Ethiopia.

Name of school	N	Seropositive (%)	Seroprevalence % (95% CI)	RT-PCR positive (%)
**Cecilia (Alamura Branch)**	35	18	51.4 (35.6, 67.0)	0 (0)
**Nigist Fura**	63	32	50.8 (38.8, 62.7)	1 (1.5)
**Ethio Parent**	63	34	54.0 (41.8, 65.7)	0 (0)
**Misrak Chora**	89	45	50.6 (40.4, 60.7)	1 (1.1)
**Addis Ketema**	81	46	56.8 (45.9, 67.0)	0 (0)
**Tula**	88	42	47.7 (37.6, 58.0)	0 (0)
**Total**	419	217	51.8 (47.0, 56.5)	2 (0.5)

### 2.2 Study design and participants

The study employed a prospective study design where data and biological samples were collected at two time points: baseline (01-08 December 2021) and follow-up after 5 months (09-18 May 2022) (**Figure 1**). The study was conducted to detect a change in seroprevalence of SARS-CoV-2 antibodies at two-time points and compare with levels of BNT vaccine-induced antibodies at 4-6 weeks after one dose or two doses of immunization. While the first dose was administered from 01 to 13 January 2022, the second dose was administrated from 22 March to 25 April 2022, with an average interval of 3 weeks (**Figure 1**). During this study period, COVID-19 vaccination was given to schoolchildren whose age was 12 years and above.

### 2.3 Data and sample collection

Participant schools were invited via an initial letter from Sidama Regional Public Health Institute and Hawassa School authorities. The main objectives of the study were described to the students during the flag and national ceremony, and further explanation was given during data collection (before specimen collection). Children who attended the selected schools were enrolled voluntarily and randomly after their parents/guardians agreed. The recruitment of participating children was carried out by trained health personnel. After obtaining informed consent and/or assent from each study participant’s guardian and/or participant, a structured questionnaire-based interview was administered to collect sociodemographic and clinical data. The interview was conducted in a private setting to ensure the safety and confidentiality of participants. Five ml of saliva and venous blood sample were taken from every participant schoolchild at each examination time point (**Figure 1**). The saliva sample was collected and processed as outlined previously (20).

### 2.4 Measurement of antibodies

Since the current mRNA-based COVID-19 vaccine is a spike protein-based vaccine (21), we used spike RBD IgG ELISA to assess the magnitude of BNT vaccine-induced humoral immune responses. Levels of anti-SARS-CoV-2 RBD-specific antibodies were analyzed with an in-house ELISA (22). Briefly, purified recombinant SARS-CoV-2 antigens based on Wuhan Hu-1 isolate (provided by the BEI Resources Repository, National Institute of Allergy and Infectious Diseases, USA) were coated on 96-well plates (1.0 μg/mL of RBD). Serum samples were diluted at 1:200 according to the optimized protocol in our previous study (22). IgG level was determined with absorbance or optical density (OD) measurement at 450 nm wavelength. Thresholds to determine seropositivity for anti-RBD IgG ELISA were calculated as previously described (22). The serum or plasma samples used for the threshold calculation of both anti-RBD IgG and IgA ELISAs have been described previously (17, 21). Each serum sample was run/tested in duplicate.

In order to evaluate the multi-variant cross-reactivity of SARS-CoV-2 infection- and BNT vaccine-induced humoral immunity, the levels of binding IgA antibodies to the recombinant RBD of the SARS-CoV-2 Wuhan-Hu-1 strain (Catalog No. NR-52307), as well as AY.2 Lineage of Delta (Catalog No. NR-55711), and B.1.1.529 BA.2 Lineage of Omicron (Catalog No. NR-56548) variants were determined by ELISA, as previously described, with a slight modification (22). All these three recombinant RBD antigens were obtained through the BEI Resources Repository. Because of the unavailability of secondary antibodies, we were unable to look at the profiles of the binding IgG and IgM antibodies against RBD variants.

Besides the detection of anti-RB IgG antibodies, saliva samples that were collected at baseline and during the follow-up studies were tested using a BGI real-time fluorescent reverse transcription (RT)-PCR kit as described previously (20).

### 2.5 Data analysis

Descriptive statistics and the actual number of cases were used to describe frequency outputs for categorical variables. We compared the level of anti-RBD IgG antibody responses between vaccinated schoolchildren (one-dose versus two-dose recipients) with and without prior SARS-CoV-2 exposure using the Wilcoxon-Mann-Whitney (WMW) matched or unmatched pairs test. To see the influence of age, sex, and self-reported history of COVID-19-like symptoms on the levels of SARS-CoV-2 infection-induced and vaccine-induced anti-RBD IgG antibodies, further analyses were done after stratifications. We visualized and studied the longitudinal anti-RBD IgG antibody response and the comparative levels of binding IgA antibodies to RBD of SARS-CoV-2 variants using locally weighted scatterplot smoothing, and the Wilcoxon-Mann-Whitney (WMW) test in GraphPad Prism Version 9.1, and *p*-values equal to or < 0.05 were considered to be statistically significant.

### 2.6 Ethical consideration

The study protocol involving human subjects was reviewed and approved by the Institutional Review Board (IRB) of the College of Medicine and Health Science, Hawassa University with the approval number IRB/009/14.

## 3 Results

### 3.1 Demographic characteristics of participants at enrolment in December 2021

**Table 1** summarizes the number of enrolled schoolchildren at baseline per each participating school. The baseline characteristics of a total of 419 schoolchildren who provided saliva and blood samples are presented in **Table 2**; **Figure 1**. Of these, 245 (58.3%) were female. The median age was 16 years (interquartile range [IQR] 13-16), 263 (62.8%) of them were in the age group of 15-19 years and 141 (33.8%) of them were in the age group of 10-14 years while 15 (3.6%) were in the age group of 7-9 years. At the follow-up visit, a total of 300 schoolchildren provided saliva and blood samples (**Figure 1**), of which 179 (59.7%) were female; the median age was 15 years (interquartile range [IQR] 12-17),174 (58.0%) were in the age group of 15-19 years and 80 (26.7%) were in the age range of 12-14 years (**Table 2**).

**Table 2:** Baseline characteristics of study participants (n = 419) and SARS-CoV-2 IgG antibody positivity rate before BNT vaccination by age, sex, and COVID-19-like symptoms.

Variable group		N (%)	Negative	Positive	Seroprevalence% (95% CI)
Sex	Male	174 (41.7)	82	92	52.9 (45.5-60.2)
	Female	245 (58.3)	120	125	51.0 (44.8-57.2)
Age	7-9	15 (3.6)	6	9	60.0 (35.7-80.2)
	10-14	141 (33.8)	72	69	49.0 (40.8-57.1)
	15-19	263 (62.6)	123	140	53.2 (47.2-59.2)
Presence of COVID-like symptoms	Yes	165 (39.3)	3	162	98.2 (94.5-99.6)
	No	254 (60.7)	199	55	21.3 (16.7-26.8)
Had positive via RT-PCR testing at enrolment	Yes	2 (0.5)	1	1	50.0
	No	417 (99.5)	201	216	51.3
Overall		419	202	217	51.8. (47.–56.5)

### 3.2 RT-PCR

Of the 419 schoolchildren enrolled at baseline, only 2 (0.5%) were positive for the RT-PCR test against SARS-Cov-2. Anti-RBD IgG antibodies were detected in one of the two RT-PCR test-positive cases both at both enrollment and follow-up. On the other hand, SARS-CoV-2 was detected in 10 (3.3%) of the 300 schoolchildren who continued in the follow-up study and submitted saliva. Interestingly, one child showed a positive RT-PCR test against SARS-CoV-2 both at the baseline and follow-up, indicating either a re-infection or persistence of viral shedding. Of the 10 RT-PCR-positive cases, seven had detectable anti-SARS-CoV-2 IgG antibodies at the 5-month follow-up time. The absence of detectable anti-RBD IgG in those three RT-PCR-positive schoolchildren may indicate a recent SARS-CoV-2 infection.

### 3.3 Seroprevalence of SARS-CoV-2 infection-induced anti-RBD IgG antibodies at baseline and 5-month follow-up

At baseline, 419 schoolchildren were surveyed in six schools (**Table 1**). However, a significant proportion of schoolchildren (28.4%) were lost to follow-up visits due to an unwillingness to provide a second blood sample, school absenteeism during the follow-up, relocation to another school, or school dropout (**Figure 1**).

Of the 419 children enrolled at the baseline, 217(51.8%) had RBD-specific SARS-CoV-2 serum antibodies at enrolment. The anti-RBD IgG antibodies seroprevalence rate was shown not to vary by the school location, whether it was located in urban or rural areas. The pooled anti-RBD IgG seroprevalence rate was 51.8% (95% CI, 47.0%–56.5%) at baseline, which was conducted in a period after the third post-Delta variant wave of the country (**Table 1**).

As shown in **Figure 1**, only 300 children were available to provide the second-round saliva and blood samples, of whom, 101 received either one dose or two doses of the BNT vaccine while the remaining 199 remained unvaccinated. Of the small sample size (n=90) of schoolchildren who were seronegative at baseline and had remained unvaccinated at the 5-month follow-up, the period during a post-Omicron variant wave, 62.2% (56/90) of them underwent a seroconversion (**Table 3**; **Figure 1**). We, therefore, noted over 10% seroprevalence difference between the baseline study (post-Delta wave) and the 5-month follow-up study (the first post-Omicron wave).

**Table 3:** Proportion (n, %), of participants (n=300) at five-month follow-up by vaccination of status, number of vaccine doses received, and positivity for the BNT vaccine-induced humoral immunity (anti-receptor-binding domain) with and without pre-existing anti-RBD IgG antibodies.

S/N	Number of BNT vaccine doses received at study follow-up	Total (*n*)	Mount detectable infection-induced anti-RBD IgG before vaccination at baseline	Total(*n*)	Sero-converted (*n*)	Sero-reverted(*n*)	Sero-persist(n)	Nonresponsive/ Remained seronegative (n)
2	None (unvaccinated)	199	Yes	109	–	4	105	–
			No	90	56	–	–	34
2	Received one dose of a two-dose schedule	80	Yes	44	–	–	44	–
			No	36	32	–	–	4
3	Received two doses of a two-dose schedule	22	Yes	11	–	1	10	–
			No	12	10	–	–	2

**n** represents the total number of participating schoolchildren; “-” implies the number of children in that particular row for a particular variable defined in the column is null.

At the 5-month follow-up, serum anti-RBD IgG antibodies persistence was observed in 96.3% (105/109) of unvaccinated children (**Table 3**; **Figure 1**).

### 3.4 Anti-SARS-CoV-2 infection-induced seroprevalence, according to sex, age, and history of COVID-19-defining symptoms

Although using a small sample size, analyses of seroprevalence by age at baseline revealed a significant proportion of children in the age group of 7-9 years mounted detectable anti-RBD IgG antibody levels than children in age groups of 10-14 years and 15-19 years (**Table 2**). Although a higher portion of female (58.3%) schoolchildren than males were recruited in this study, a slightly increased seroprevalence was observed among male schoolchildren (**Table 2**). Importantly, a sizeable proportion (74.7%) of the participating schoolchildren who had detectable anti-SARS-CoV-2 antibody levels had a history of COVID-19-defining symptoms (**Table 2**). Additionally, we noted the absence of a statistically significant (*p* > 0.05) association between anti-RBD IgG seropositivity and socioeconomic variables, such as family size, and education level of the schoolchildren’s father and mother (**Table S1**).

### 3.5 Seroprevalence of the BNT vaccine-induced anti-RBD IgG antibodies

**Figure 2:**
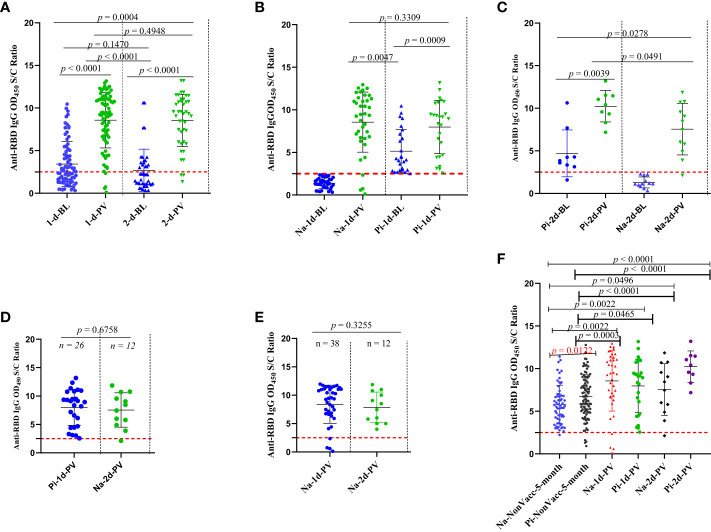
Comparison of the natural infection- and BNT vaccine-induced humoral immunity response in vaccinated and unvaccinated schoolchildren with and without baseline pre-existing anti-RBD IgG antibodies against Wuhan-RBD, A-F. Levels of anti-RBD IgG comparison between **(A)** schoolchildren (n = 80) who received one dose versus those schoolchildren (n = 21) who received two-doses of the BNT vaccine; **(B)** schoolchildren who received one dose of the BNT vaccine with and without a baseline pre-existing anti-RBD IgG antibodies; **(C)**: schoolchildren who received two doses of the BNT vaccine with and without a baseline pre-existing anti-RBD IgG antibodies; **(D)**: schoolchildren (n = 26) who received one dose of BNT vaccine with a baseline pre-existing anti-RBD IgG antibodies versus SARS-CoV-2-infection-naïve schoolchildren (n = 12) who received two doses; **(E)**: schoolchildren (n = 38) who received one dose of the BNT vaccine with baseline pre-existing anti-RBD IgG and schoolchildren (n = 12) who received two doses of the BNT vaccine without a baseline pre-existing anti-RBD IgG antibodies: **(F)**: unvaccinated schoolchildren with and without anti-RBD IgG antibodies at baseline or 5-month follow-up versus schoolchildren who received one or two doses of the BNT vaccine with or without a baseline pre-existing anti-RBD IgG. The levels of serum anti-RBD IgG antibodies were measured using serum samples diluted at 1:200 in an in-house indirect ELISA. The OD_450_ S/C ratio value on the y-axis represents the ratio of the sample OD_450_ nm to the average mean OD_450_ nm of the negative controls. The horizontal broken red line shows the cut-off value (= 2.5) for the in-house ELISA test. A Wilcoxon-Mann-Whitney (WMW) matched or unmatched pairs test was performed to compare differences between the two groups. A *p-value* of < 0.05 indicates the occurrence of a statistically significant difference in anti-RBD IgG levels between any two comparison groups while ns or *p* > 0.05 indicates a statistically insignificant difference. 1d, 2d, and NonVacc represent schoolchildren who received a single dose, two doses of the BNT vaccine, and neither a first dose nor a second dose, respectively; BL, PV, and 5-month indicate that serum samples were collected before schoolchildren’s vaccination (baseline), postvaccination (after receiving one or two doses of the BNT vaccine), and at the 5-month follow-up visit from unvaccinated children, respectively; Na and Pi indicate being SARS-CoV-2 infection naïve (negative for anti-RBD IgG serological test at baseline) and having a prior SARS-CoV-2 infection (positive for anti-RBD IgG serological test at the baseline), respectively.

Of a total of 300 schoolchildren who were available at the five-month follow-up, 101 had received either one dose (n = 80) or two doses (n = 21) of the two-dose BNT vaccine schedule while 199 schoolchildren remained unvaccinated (**Table 3**). At the enrolment (baseline) before vaccination, nearly 50% (51/101) of schoolchildren in the vaccinated group mounted a detectable anti-RBD IgG antibody level (**Table 3**; **Figure 1**). No marked difference was seen between those schoolchildren receiving one dose and those schoolchildren receiving two doses in terms of the proportion of schoolchildren with a prior SARS-CoV-2 infection and the mean levels of infection-induced RBD IgG antibody response (**Figure 2**). However, 4 to 6 weeks after BNT vaccination, 95.1% (96/101) of the vaccinated schoolchildren, irrespective of their anti-RBD IgG serostatus at enrolment, mounted detectable antibody levels. Interestingly, 90.2% (46/51) of SARS-CoV-2-infection-naïve-vaccinated schoolchildren had detectable anti-RBD IgG antibodies. Of those five schoolchildren who were non-responsive to the BNT vaccine, four had received one dose while the remaining one had received two doses. By contrast, 100% (n = 52) of the BNT-vaccinated schoolchildren with prior SARS-CoV-2 infection had detectable anti-RBD IgG antibodies at 4-5 weeks postvaccination, irrespective of the number of doses they had received (**Figure 1**; **Table 2**).

### 3.6 Comparison of SARS-CoV-2 anti-RBD IgG antibodies levels in different groups: one-dose versus two-dose recipients; prior infection versus naïve vaccinated recipients

**Figure 3:**
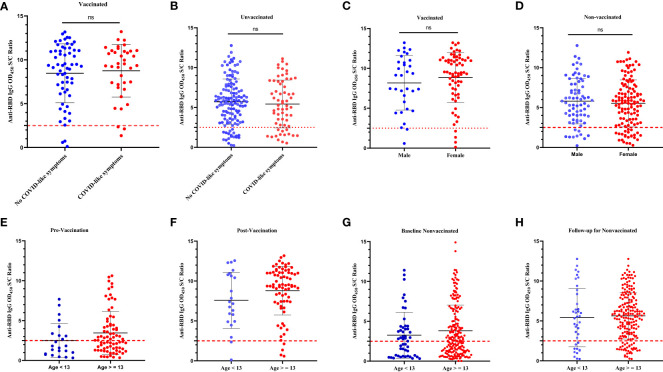
Comparison of the levels of infection-induced and BNT vaccine-induced anti-RBD IgG responses by the self-reported history of COVID-19-defining symptoms, sex, and age. The levels of anti-RBD IgG in **(A)**: vaccinated schoolchildren with COVID-19-defining symptoms versus vaccinated schoolchildren without COVID-19-defining symptoms; **(B)**: unvaccinated schoolchildren with COVID-19-defining symptoms versus unvaccinated schoolchildren without COVID-19-defining symptoms; **(C)**: male vaccinated schoolchildren versus female vaccinated schoolchildren; **(D)**: male unvaccinated versus female unvaccinated schoolchildren; **(E)**: schoolchildren of age < 13 years at prevaccination versus schoolchildren of age ≥ 13 years at prevaccination; **(F)**: schoolchildren of age < 13 years at postvaccination versus schoolchildren of age ≥ 13 years at postvaccination; **(G)**. unvaccinated schoolchildren of age < 13 years at baseline versus unvaccinated schoolchildren of age ≥ 13 years at baseline; **(H)**: unvaccinated schoolchildren of age < 13 years at 5-month follow-up versus unvaccinated schoolchildren of age ≥ 13 years at 5-month follow-up. A Wilcoxon-Mann-Whitney (WMW) matched or unmatched pairs test was performed to compare differences between the two groups. For all panels A-H, the *p-value*> 0.05. ns = *p* > 0.05 indicates a statistically insignificant difference. The OD_450_ S/C ratio value on the y-axis represents the ratio of the sample OD_450_ nm to the average mean OD_450_ nm of the negative controls. The broken black line represents the cut-off value (2.5).

**Table 3** summarizes the number of participants by vaccination of status, the number of vaccine doses received, and positivity for SARS-CoV-2 natural infection- and BNT vaccine-induced anti-RBD IgG antibodies at the 5-month follow-up time. In general, we did not notice differences (*p* > 0.05) in the levels of the BNT vaccine-induced anti-RBD IgG antibody response between the schoolchildren (n = 80) who received one dose and those schoolchildren (n = 21) who received two doses of the BNT vaccine (**Figure 2A**). When we compared the levels of vaccine-induced anti-RBD IgG response among those who received one dose of the BNT vaccine, schoolchildren with pre-existing anti-RBD IgG antibodies generated higher levels of anti-RBD IgG antibodies than schoolchildren who had no detectable pre-existing anti-RBD IgG antibodies although the difference was not statistically significant (*p* = 0.3) (**Figure 2B**). However, this difference was statistically significant (p < 0.05) when comparing the levels of BNT-induced anti-RBD IgG antibody response between vaccinated schoolchildren with prior infection who received two doses and SARS-CoV-2-infection-naïve schoolchildren who received two doses (**Figure 2C**). Interestingly, we found no difference (*p* > 0.5) in the mean levels of anti-RBD IgG antibody response between the schoolchildren (n = 26) with prior SARS-CoV-2 infection who received one dose of the BNT vaccine and the SARS-CoV-2-infection-naïve schoolchildren (n = 12) who received two doses (**Figure 2D**). Similarly, we observed no marked difference between single-dose (n = 38) and two-dose (n = 12) BNT vaccine-induced anti-RBD IgG antibody levels in SARS-CoV-2-infection-naïve schoolchildren (**Figure 2E**). As expected, we noted a marked significant difference (*p* < 0.05) between the levels of post-SARS-CoV-2 infection- and BNT vaccine-induced anti-RBD IgG antibody responses, irrespective of the number of doses received (**Figure 2F**). More importantly, age, sex, and the presence of COVID-19-defining symptoms did not impact the levels of vaccine-induced anti-RBD IgG antibody response (**Figures 3A–H**).

### 3.7 Levels of binding IgA antibodies to SARS-CoV-2 RBD variants in vaccinated and unvaccinated schoolchildren

**Figure 4:**
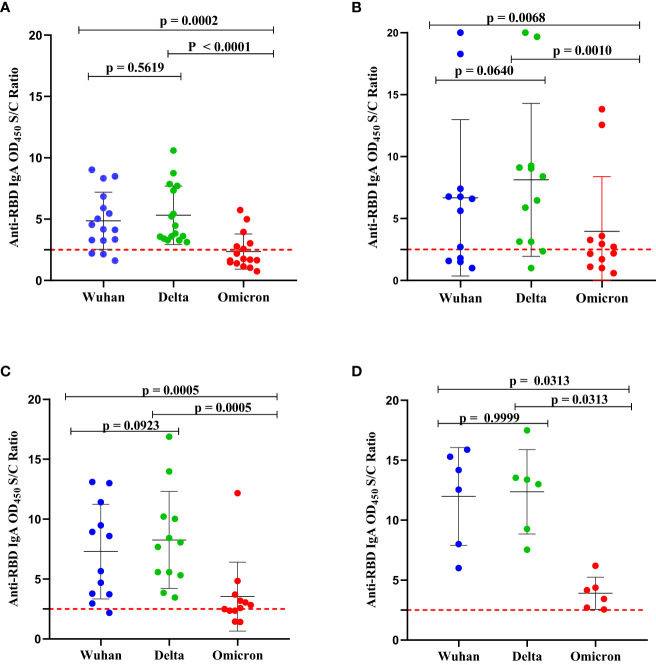
Comparison of the levels of binding IgA antibodies to the RBD SARS-CoV-2 of Wuhan strain, Delta (AY.2 lineage) variant, and Omicron (B.1.1.529 BA.2 lineage) variant. **(A)** SARS-CoV-2-infection-naïve unvaccinated schoolchildren (n = 13); **(B)** unvaccinated schoolchildren with prior (baseline) SARS-CoV-2 infection (n = 8); **(C)** SARS-CoV-2-infection-naïve BNT-vaccinated schoolchildren (n = 11) and **(D)** BNT-vaccinated schoolchildren with prior (baseline) SARS-CoV-2 infection (n = 6). The antibody levels were determined in serum samples collected at the 5-month follow-up using an in-house indirect ELISA. The OD_450_ value on the y-axis was calculated as the ratio of the optical density (OD) of the sample to the mean ODs of negative controls. An antibody level (OD_450_ nm S/C ratio) below 2.5 was interpreted as negative. A Wilcoxon-Mann-Whitney (WMW) matched or unmatched pairs test was performed to compare the difference between the two groups. *p-value* < 0.05 indicates the presence of a statistically significant difference while *p* > 0.05 indicates a statistically insignificant difference. The broken line represents the cut-off value, of 2.5.

Due to the unavailability of secondary (HRP-conjugated) anti-human IgG antibodies, we determined and compared the levels of natural SARS-CoV-2 -and BNT vaccine-induced serum IgA antibodies to Delta-RBD, Omicron-RBD, and Wuhan-RBD using an in-house indirect ELISA. Additionally, due to the limited availability of Delta-RRBD and Omicron-RBD, we used randomly selected serum samples (n = 38) collected at the 5-month follow-up, and all were confirmed to have detectable anti-RBD IgG antibodies. The randomly selected serum panel was comprised of four different groups of children:

Group 1: SARS-CoV-2-infection-naïve unvaccinated schoolchildren (n = 13) (**Figure 4A**);
Group 2: unvaccinated schoolchildren with prior (baseline) SARS-CoV-2 infection (n = 8) (**Figure 4B**);
Group 3: SARS-CoV-2-infection-naïve BNT vaccinated schoolchildren (n = 11) (**Figure 4C**); and
Group 4: BNT-vaccinated schoolchildren with prior (baseline) SARS-CoV-2 infection (n = 6) (**Figure 4D**).

The pattern of binding IgA antibodies levels to Delta-RBD, Wuhan-RBD, and Omicron-RBD appeared to be similar in the four groups (**Figures 4A–D**) described above. A significantly higher level of binding IgA antibodies to both Wuhan-RBD and Delta-RBD compared to Omicron-RBD (*p* < 0.001) was observed. Although the difference was not statistically significant, we noted slightly higher levels of natural infection- and BNT vaccine-induced binding IgA antibodies to Delta-RBD than to Wuhan-RBD (**Figures 4A–C**). When we compared anti-Omicron-RBD IgA seropositivity among the four groups, 38.4%, 75%, and 63.6% of the schoolchildren in group 1, group 2, and group 3, respectively, had detectable IgA antibodies to Omicron-RBD, above the assay’s lower detection limit (**Figures 4A–C**). By contrast, all six (100%) vaccinated schoolchildren with prior infection SARS-CoV-2 infection in group 4 had detectable IgA antibodies to Omicron-RBD (**Figure 4D**).

### 3.8 Reactogenicity and safety

Although assessing reactogenicity was not the primary objective of the present study, the BNT vaccine was found to be well-tolerated by all 101 vaccine recipients with no single case of a serious adverse event. The majority (58.4%) of the vaccine-recipient schoolchildren had self-reported pain and tenderness at the vaccine injection site accompanied by severe headache and fatigue after receiving one. However, all these symptoms were resolved within 48 hours postvaccination. None of them reported side effects within 48 hours of receiving the second dose (data not shown).

## 4 Discussion

As RBD is the major target for neutralization antibodies (nAbs), antibody levels to this fragment of spike protein are a good alternative to predict serum neutralization function, especially in resource-constrained countries like Ethiopia (16). We specifically used an in-house anti-RBD IgG ELISA to assess natural infection- and BNT vaccine-induced antibody responses in schoolchildren in Hawassa, Ethiopia. Interestingly, schoolchildren were shown to mount an adequate anti-RBD IgG antibody response both to natural infection and to BNT vaccination. To the best of our knowledge, this is the first data describing both SARS-CoV-2 infection- and BNT vaccine-induced humoral immunity in schoolchildren in Ethiopia.

Although children are generally believed to have a lower burden of symptomatic COVID-19 compared to adults, a sizeable portion (74.7%) of the participating schoolchildren who had detectable anti-SARS-CoV-2 antibody levels had a history of COVID-19-defining symptoms. Our finding is consistent with the post-Delta and Omicron reports of increased risk of symptomatic infection and mortality in children (4) and warrants the urgent need to administer CoVID-19 vaccines to children (10). Our local real-world data on immunogenicity and reactogenicity of the BNT vaccine along with a previous study from South Africa (13) would be useful to circumvent the low vaccine intake in African children due to the misconception on safety and efficacy of COVID-19 vaccines (23). More importantly, vaccination of children will more likely prevent schoolchildren from symptomatic infection, thereby broadening community protection and preventing long COVID and will allow schools to resume their duties (10).

The observed high (51.8%) SARS-CoV-2 seroprevalence in schoolchildren at the beginning of December 2022 is inconsistent with most studies that reported lower seroprevalence in schoolchildren as compared to adults (7). However, this finding is consistent with our previous study that revealed the occurrence of high seroprevalence (44.8%) among healthcare workers in Hawassa Hospital (22). It is important, however, to note that the present study was conducted 10 months later than our previous seroprevalence study. At the 5-month follow-up (during the post-Omicron wave (19)), we observed a high (62.2%) seroprevalence (seroconversion) rate among unvaccinated schoolchildren, which is more likely linked to the spread of the highly transmissible Omicron variants. Our two time point seroprevalence findings are consistent with a systematic review report by Naeimi et al. (5), in which SARS-CoV-2 seroprevalence of 17.2% (0.01–58.4%) was observed in the pre-Omicron wave and 66.1% (60.53–71.4%) in a post-Omicron wave, in the African region. Poor adherence to COVID-19 nonpharmaceutical mitigation strategies during school reopening (data not shown) as well as a poor vaccination rate would likely be among the predominant reasons for higher SARS-CoV-2 seroprevalence estimates in the participating schoolchildren as previously reported by Naeimi et al (5). These observed higher seroprevalence rates in schoolchildren could also be linked to the higher sensitivity of the in-house ELISA we used as a serodiagnosis test (22).

In the present study, the risk of seroprevalence was found to be associated with the presence of COVID-19 symptoms yet independent of age, sex, school location in urban or rural areas, and socio-economic variables, such as family size, and education level of the children’s father and mother. This is inconsistent with a pre-Omicron seroprevalence study conducted among a cohort of children and teenagers in Montreal, Canada, where parents’ low education and higher household density were found to be associated with an increased risk of seropositivity (24). This difference may be due to the minimal social inequalities in our study areas as well as a similar poor adherence to non-pharmaceutical interventions in the community and as a consequence, the children might have had equal risk of exposure to SARS-CoV-2 infection.

With waning immune protection over time against emerging SARS-CoV-2 variants, it is essential to understand the duration of infection- and vaccine-induced immunity or both to establish vaccine strategies. The second goal of the present study was therefore to obtain real-world local data on the immunogenicity BNT vaccine in schoolchildren in Ethiopia. The BNT vaccine was shown to induce RBD-specific IgG antibodies, which is a proxy marker for measuring the magnitude of the neutralization antibodies. Of the 101 vaccinated schoolchildren who received either one or two doses of the BNT vaccine, we noted high immunogenicity of the BNT vaccine in schoolchildren, inferred from a 90.2% vaccine-induced seroconversion rate among the SARS-CoV-2-infection-naïve schoolchildren. This observation is not a surprise as all 5 were non-responsive but one had received only one dose of vaccine which might have happened due to the rapid waning of the single-dose vaccine-induced immunity. A similar seroreversion rate (94.2%) with anti-RBD antibody levels below the seropositivity threshold was previously reported in BNT-vaccinated adults in the first 3 months post-vaccination (25).

In the present study, the natural SARS-CoV-2 infection-induced serum anti-RBD IgG persistence rate at the 5-month follow-up was 96.3% (105/109) in the unvaccinated children, indicating a relative long-term protective immunity after SARS-CoV-2 infection in children. More importantly, we noticed a robust anti-RBD IgG antibody response in schoolchildren with prior infection and who received one dose of the BNT vaccine, comparable to the infection-naïve schoolchildren who received two doses. Based on our findings, a single dose of BNT vaccination might be sufficient to generate a breadth of humoral immunity against SARS-CoV-2 variants in recovered children with pre-existing anti-SARS-CoV-2 infection, particularly in settings where vaccine supply shortage is a constraint. Consistent with this finding, recent studies demonstrate that infection-induced memory B cells (MBCs) have a better antigen-binding capacity and generate secondary MBCs, undergo more affinity maturation, and produce more robust secondary responses than vaccine-induced primary MBCs (24–26). In addition, the predicted median time to breakthrough infection following mRNA vaccination, including BNT, has been shown to be longer than the median time to breakthrough infections following viral vector vaccination (27). Further, unlike adenovirus-vector COVID-19 vaccines, vaccination with BNT has been shown to reactivate pre-existing, cross-reactive T-cell immunity, particularly in children (28).

Like adults, the administration of two doses of the BNT vaccine is required to generate an optimal immune response in children. Furthermore, the dosing interval has been shown to influence the vaccine-induced humoral response (27–30). In the present study, we did not observe a noticeable difference in the levels of the BNT vaccine-induced anti-RBD IgG antibody response between the SARS-CoV-2-infection-naive schoolchildren who received one dose and those who received two doses. This observation could be because more schoolchildren (n=38) who received one dose than those who received two doses (n=12) were included in our analysis. However, the short dosing interval (< 4 weeks) between the first dose and the second dose could be a plausible reason. In support of the latter, a delayed administration of the second dose of the BNT vaccine up to 12 weeks has been shown to produce strong and more robust antibodies than early administration (29–32).

As of February 2022, Ethiopia had experienced four distinct waves of SARS-CoV-2 infections. Although the responsible variant for the first (May to November 2020) wave remains unknown, the second (January to June 2021), third (August to November 2021), and fourth (December 2021 to February 2022) waves were fueled by the Alpha, Deta, and Omicron variants, respectively (19). Since our first and second-round serum sample collections were succeeded by the third and fourth COVID-19 pandemic waves in Ethiopia, respectively, we compared the levels of binding IgA antibodies to RBDs of the SARS-CoV-2 Delta and Omicron variants as well as to Wuhan-RBD in unvaccinated and BNT-vaccinated schoolchildren. Although not statistically significant, relatively higher levels of binding IgA antibodies to Delta-RBD compared to Wuhan-RBD were observed in the majority of unvaccinated and BNT-vaccinated children who had pre-existing anti-RBD IgG antibodies (**Figure 4**), indicating most of these schoolchildren were likely to have had SARS-CoV-2 infection with the Delta variant. In comparison to Delta-RBD and Wuhan-RBD, the levels of binding IgA antibodies to Omicron-RBD were found to be significantly lower, even below the threshold for some schoolchildren. This finding is consistent with the finding that Omicron-RBD contains 15 mutations while the Delta-RBD contains only two mutations as compared to the wild-type, Wuhan-RBD (33). Also, the detection of low levels of IgA antibodies to the Omicron-RBD in SARS-CoV-2-infection-naïve BNT-vaccinated schoolchildren is not a surprise given that these children received the first generation of mRNA-based vaccine, developed based on stabilized spike protein sequence homologous to the wild-type (D614) Wuhan strain (34) and Omicron-RBD bears 15 mutations. Interestingly, we noted a broader IgA antibody cross-reactivity to the RBDs from SARS-CoV-2 variants in vaccinated schoolchildren with prior SARS-CoV-2 infection than in vaccinated SARS-CoV-2 infection naive schoolchildren, supporting the superiority of hybrid immunity (35).

Besides its immunogenicity, the BNT vaccine was well-tolerated among vaccinated children with no serious adverse event despite the majority of vaccinees having self-reported pain and tenderness at the injection site accompanied by fever and headache that resolved within 48 hours postvaccination. Although severe COVD-19 and associated death are rare in children, long-COVID, a post-acute sequelae of SARS-CoV-2 infection, is not an uncommon complication in children (36) and could be considered as an additional argument to increase vaccine uptake in schoolchildren. Our data, therefore, provide local evidence on the safety/reactogenicity profiles and immunogenicity of the BNT vaccine among Ethiopian schoolchildren and will help to accelerate the COVID-19 vaccine uptake and coverage among school-aged children in Ethiopia.

## 5 Limitations of the study

Despite its strength, our study has several limitations. First, due to budget constraints, we did not collect second-round postvaccination serum samples to evaluate the dynamics of BNT vaccine-induced anti-RBD IgG antibody response over time. Second, due to the reagents’ unavailability, we did not assess infection-induced and BNT vaccine-induced T-cell responses. Third, again due to budget constraints, we were not able to perform either virus or pseudovirus neutralization assays. However, we measured the level of anti-RBD IgG antibody response, which is a good proxy marker for nAbs. The fourth limitation is the low sample size in the postvaccination analysis due to the significant unwillingness or loss of schoolchildren at the follow-up visit. However, the enrolment of a total of 101 schoolchildren before and after BNT vaccination was acceptable to draw a conclusion regarding the immunogenicity and reactogenicity of the BNT vaccine in schoolchildren between the ages of 12 and 19 years old. The fifth limitation is that we did not convert the measured level of binding anti-RBD IgG and IgA antibodies into a unit of 1000 binding antibody units (BAU) per mL as described by Knezevic et al (37) due to the unavailability of a reference plasma sample (research reagent 20/130) distributed by National Institute for Biological Standards and Control (NIBSC), UK, a WHO collaborative center.

## 6 Conclusions

In conclusion, our findings from serological evidence indicated that, although there were significant increases in SARS-CoV-2 seroprevalence in children from 51.8% in the pre-Omicron wave to 62.2% in the post-Omicron wave, a significant proportion of schoolchildren in Ethiopia remained unvaccinated. Despite the small sample size of study participants, the BNT vaccine is shown to be immunogenic and safe for schoolchildren. These findings emphasize an urgent need to expand vaccination coverage for children and adolescents, particularly in school settings. Single-dose administration of the BNT vaccine may be considered for children who have had a prior SARS-CoV-2 infection when a shortage of vaccine supply is a limiting factor to administer two doses of the BNT vaccine irrespective of their serostatus.

Although it is from a small sample size, our data on the profile of binding IgA antibodies to RBD of SARS-CoV-2 variants in both BNT-vaccinated and unvaccinated schoolchildren support the superiority of hybrid immunity in providing a multivariant cross-reactive humoral immune response. However, future investigations in large longitudinal cohorts of SARS-CoV-2-naïve and COVID-19-recovered children receiving the BNT vaccine, with the inclusion of more RBD of the SARS-CoV-2 variants circulating in Ethiopia and two more immunoglobulin classes (IgM and IgG) are needed for a better understanding of the kinetics, breadth, and durability of vaccine-, natural infection- or both (hybrid)-induced humoral immunity in children.

## Data availability statement

The original contributions presented in the study are included in the article/**Supplementary Material**. Further inquiries can be directed to the corresponding author.

## Ethics statement

The study protocol involving human subjects was reviewed and approved by the Institutional Review Board (IRB) of College of Medicine and Health Science, Hawassa University with an approval number IRB/009/14. Written informed consent to participate in this study was provided by the participants’ legal guardian/next of kin.

## Author contributions

YM, TG, AMu, and AMi conceptualized the study. YM, WT, ET, and AG contributed to the acquisition of the funding. YM, TG, AMu, AMi, WT, ET, and AG contributed to the design and supervision of the study. WT organized the database. TG wrote the first and final draft of the manuscript and YM contributed to the drafting of the manuscript. TG analyzed and visualized the data. TG, AMu, and AMi contributed to the acquisition of laboratory reagents. AlA contributed to data acquisition and collection and processing of saliva and blood samples. DF and AyA performed testing of the serum samples in ELISA. All authors contributed to the article and approved the submitted version.
