# Supplementary material for: SARS-CoV-2 Infection-and mRNA Vaccine-induced Humoral Immunity among Schoolchildren in Hawassa, Ethiopia
Source: Front Immunol. 2023 Jun 15;14:1163688. doi: 10.3389/fimmu.2023.1163688 (PMC10308774; doi:10.3389/fimmu.2023.1163688)
Supplement: Supplementary file 1 [file Table_1.docx]

Table S1. Schoolchildren’s bassline anti-SARS-CoV-2 seropositivity by socio-demographic factors

| **Variable** | **n** | **Seropositive** | **%** | ***p*-value** |
| --- | --- | --- | --- | --- |
| **Family size** | | | | |
| ≤ 3 | 43 | 21 | 48.8 | Ref. |
| 4-5 | 163 | 82 | 50.3 | 0.95 |
| ≥ 6 | 206 | 112 | 54.4 | 0.83 |
| **Level of children’s father’s education** | | | | |
| Illiterate | 87 | 47 | 50.0 | Ref. |
| Primary school | 97 | 46 | 47.4 | 0.47 |
| Secondary school | 152 | 85 | 60.0 | 0.62 |
| College and above | 84 | 37 | 44.1 | 0.41 |
| **Level of children’s mother’s education** | | | | |
| Illiterate | 125 | 71 | 56.8 | Ref. |
| Primary school | 23 | 11 | 47.8 | 0.48 |
| Secondary school | 97 | 48 | 49.5 | 0.65 |
| College and above | 175 | 85 | 48.6 | 0.40 |

n = a total number of children per variable; % seropositivity rate; *p*-value > 0.05 significances absence of association between seropositivity and variable; Ref. stands for reference.
